# Abruptio Placentae Caused by Hypertriglyceridemia-Induced Acute Pancreatitis during Pregnancy: Case Report and Literature Review

**DOI:** 10.1155/2018/3869695

**Published:** 2018-09-05

**Authors:** Pınar Yalcin Bahat, Gokce Turan, Berna Aslan Cetin

**Affiliations:** Department of Obstetrics and Gynecology, Kanuni Sultan Suleyman Training and Research Hospital, Istanbul Health Sciences University, Istanbul, Turkey

## Abstract

**Background:**

Hormonal effects during pregnancy can compromise otherwise controlled lipid levels in women with hypertriglyceridemia and predispose to pancreatitis leading to increased morbidity for mother and fetus. Elevation of triglyceride levels is a risk factor for development of pancreatitis if it exceeds 1000 mg/dL. Pancreatitis should be considered in emergency cases of abdominal pain and uterine contractions in Emergency Department at any stage of pregnancy. We report a case of abruptio placentae caused by hypertriglyceridemia-induced acute pancreatitis. Also, literature review of cases of acute pancreatitis induced by hypertriglycaemia in pregnancy has been made.

**Case:**

A 22-year-old woman presented to our Emergency Department, at 35 weeks of gestation, for acute onset of abdominal pain and uterine contractions. Blood tests showed a high rate of triglyceride. The patient was diagnosed with abruptio placentae caused by hypertriglyceridemia-induced acute pancreatitis. Immediate cesarean section was performed and it was observed that blood sample revealed a milky turbid serum. Insulin, heparin, and supportive treatment were started. She was discharged on the 10th day.

**Conclusion:**

Consequently, patients with known hypertriglyceridemia or family history should be followed up more closely because any delay can cause disastrous conclusions for mother and fetus. Acute pancreatitis should be considered in pregnant women who have sudden onset, severe, persistent epigastric pain and who have a risk factor for acute pancreatitis.

## 1. Introduction

Acute pancreatitis (AP) is a rare complication in pregnancy, occurring in approximately three in 10000 pregnancies [1, 2]. Hypertriglyceridemia is recognized as the third most common cause of gestational acute pancreatitis after gallstones and alcohol and occurs in about 4% of all cases [2]. An increase in plasma lipid level during pregnancy has been well documented. It is thought to represent a physiologic response to the hormonal changes; however, it is not sufficient to cause acute pancreatitis. Gestational pancreatitis due to hypertriglyceridemia usually occurs in pregnant women with preexisting abnormalities of the lipid metabolism. There are effective treatment choices during pregnancy such as dietary restriction of fat, intravenous heparin, and insulin and plasmapheresis. We report a case of abruptio placentae caused by hypertriglyceridemia-induced acute pancreatitis.

## 2. Case Report

A 22-year-old patient, Para 1, Gravida 2, presented to our Emergency Department of Gynecology and Obstetrics, at 35 weeks of gestation for acute onset of abdominal pain and uterine contraction. It was learned that the patient's history had no follow-up hypertriglyceridemia. On physical exam, her heart rate was at 100 pulses per minute, and her blood pressure was at 110/70 mm-Hg, respiratory rate 18 /min. Her abdomen was defensive. Her cervical os was dilated to 1-2 cm and minimal bleeding. The patient had mild epigastric tenderness. Decelerations were seen in pregnant cardiotocography follow-ups with abnormal abdominal pain and uterine contractions continued and simultaneous wide bleeding area (like abruptio placenta) was seen on the posterior part of placenta in ultrasound. Immediate cesarean section was performed under general anesthesia because of contraction of the tetanic type in the manual contraception. She gave birth to a healthy infant of 2980 g. Amylase, lipase, triglyceride, HDL, and LDL were studied in the patient's blood after emulsion of chylous fluid from abdomen during the cesarean section. Liver enzymes were high: ast: 241, sub. 147. It was observed that blood sample revealed a milky turbid serum. Laboratory finding included a triglyceride at 3297 mg/dl and amylase 827 U/L, lipase 1576 U/L. Abdominal ultrasound showed thickened pancreas without necrosis; acute pancreatitis compatible with diffuse edema was observed on pancreas. Biliary tract was naturally observed. Other causes of cholestasis of pregnancy, such as cholangitis, acute hepatitis, and hemophagocytic syndrome, were ruled out. Oral intake of the patient was stopped; intravenous fluid replacement therapy, antibiotherapy, proton pump inhibitor, insulin, and heparin therapy were started. She was discharged on the 10th day of treatment. Even though the patient did not have previous history of diabetes or gestational diabetes, the baby was born 4 to 3 weeks earlier. It was thought that this condition might be related to maternal hyperlipidemia for newborn's doctors.

## 3. Discussion

Acute pancreatitis (AP) is a rare complication in pregnancy. Diagnosis becomes difficult because it can interfere with the physiological findings in pregnancy. Acute pancreatitis should be considered in pregnancies with nausea, vomiting, and epigastric pain. Gallstones, hypertriglyceridemia, and alcohol especially play a role in the etiology of acute pancreatitis.

Hypertriglyceridemia is the second most common cause of acute pancreatitis in pregnancy. Diagnosis is made when the serum triglyceride is > 1000 mg/dl. Hypertriglyceridemia in pregnant patients can occur with preexisting dyslipidemia, associated with others diseases (hypertension, diabetes mellitus, and alcoholism), or without any predisposing factor. Triglycerides concentration rises gradually, 2.5-fold over prepregnancy levels, reaching a peak during the third trimester to almost twice as high value of nonpregnant value. This is thought to be due to estrogen-induced increases in triglyceride synthesis and very low-density lipoprotein secretion [29]. Therefore, AP is more common in the third trimester of pregnancy. Lipids decrease gradually postpartum to reach prepregnancy level in 6 weeks [30, 31]. Epigastric pain, spreading pain, nausea, vomiting, and distension can be seen at the beginning of the symptoms in acute pancreatitis cases. Findings of peritoneal irritation are not seen in general, especially when there is epigastric pain in mild cases as indicated by physical examination findings. In severe cases, epigastric tenderness and peritoneal irritation findings may be accompanied by ileus, fever, and tachycardia. The increase in serum amylase reaches peak values 6-12 hours after the onset of the event. The exact diagnosis of pancreatitis is based on the amylase/creatinine clearance rate. Serum lipase values also increase. Imaging methods can be used in the diagnosis of acute pancreatitis from ultrasonography, computed tomography, and magnetic resonance imaging. Ultrasound is the most appropriate method for pregnancy.

Acute pancreatitis treatment in pregnancy is similar to nonpregnant treatment of hyperlipidemia. Pregnancy pancreatitis treatment is primarily medicinal and approximately 90% of patients respond to medical treatment. Medical treatment of AP is mostly supportive. These treatments include low fat diet [32, 33], antihyperlipidemic therapy [32, 33], insulin [32, 34] (to increase lipoprotein lipase activity), heparin [33, 35] (to increase lipoprotein lipase activity), and even plasmapheresis [32, 35].

Our patient was admitted with acute onset of abdominal pain and uterine contraction to our clinics in the 35th week of gestation. She had lipid abnormality in her history, but her history had no follow-up hypertriglyceridemia. Pregnancy had induced aggravation of hypertriglyceridemia and associated pancreatitis. In addition, acute pancreatitis induced by the pregnancy was accompanied by abruptio placenta and delivery was performed with an emergency cesarean section. It was observed that blood sample revealed a milky turbid serum. We managed our patient conservatively in postoperative period. Oral intake of the patient was stopped; intravenous fluid replacement therapy, antibiotherapy, proton pump inhibitor, insulin, and heparin therapy were started. The patient's clinical condition subsequently improved.

Cases of acute pancreatitis induced by hypertrigliceridemia during pregnancy published in the literature are listed in Table 1. In the majority of published case, medical treatment was first tired. Oral intake was closed, supportive treatment started. However, pregnancy-induced pancreatitis has been mortal in some cases and has gone as far as maternal death.

Ihuang et al. performed a retrospective study on 21 pregnant women diagnosed with acute pancreatitis (AP). Patients were divided into acute biliary pancreatitis (A BP), hypertriglyceridemia-induced acute pancreatitis (HTG P), and idiopathic groups according to etiology. 95% of the patients were in the third trimester of gestation. The percentage of patients with HTGP was higher than that of ABP (48% versus 14%). The percentage of severe acute pancreatitis (SAP) in the HTGP group was higher than that in the ABP group (40.0% versus 0%). In the HTGP group, five patients given were plasma exchange therapy and five were not. According to the results of this study it was found that plasma exchange may be safe and effectively administered for HTGP patients during pregnancy with SIRS or multiple organ dysfunction syndrome (MODS) [36].

In a study by Lingyu Luo et al., they retrospectively reviewed 121 acute pancreatitis in pregnancy (APIP) cases. The correlation between APIP types, severity, biochemical parameters, and mortality was analyzed. The most common causes of APIP were gallstone and hypertriglyceridemia. Lower level of serum calcium could be used as an indicator for the severity of the APIP. According to the result s of this study it was found that the severity of APIP was associated with higher risk for neonate asphyxia and maternal and fetal death [37].

In a prospective study performed by Athyros VG et al., 17 cases of acute pancreatitis induced by hypertriglyceridemia were included in the study. These patients were followed for 42 months. In the content of the study causative conditions of HTG-induced A P were familial HTG in eight patients, HTG caused by uncontrolled diabetes mellitus in five, HTG aggravated by drugs in two (one by tamoxifen and one by fluvastatin), familial hyperchylomicronemia (HCM) in one, and lipemia of pregnancy in one. During the acute phase of pancreatitis, patients underwent standard treatment. After that, HTG was efficiently controlled with high dosages of fibrates or a fibrate plus acipimox, except for the patient with H CM, who was on a specific diet (the only source of fat was a special oil consisting of medium chain triglyceride) and taking a high dosage of acipimox. One of the patients died during the acute phase of pancreatitis with acute respiratory distress syndrome. According to the results of the study it was found that appropriate diet and drug treatment, including dose titration, of severe HTG are very effective in preventing relapses of HTG-induced AP [38].

As a result, pancreatitis can be seen in pregnancy in cases with uncontrolled hypertriglyceridemia. Patients with known hypertriglyceridemia or family history should be followed up more closely. Acute pancreatitis should be considered in pregnant women who have sudden onset, severe, persistent epigastric pain and who have a risk factor for acute pancreatitis.

## Figures and Tables

**Table 1:** Case literatures of acute pancreatitis induced by hypertriglyceridemia during pregnancy.

First Author	Year	Age	G/P	Birth	Medication	Other	Mode BW	Indication	Laboratory *∗*	After Treatment *∗∗*
Billion JM [3]	1991	32		35	TPN					

Achard JM [4]	1991				Two Lipaphereses					

Perrone G [5]	1996	37		35	Diet, Gemfibrozil					

İbrahim Bildirici [6]	2002	26	G2P2	24	Insulin, Plasmapheresis		C/S	Fetal Distress (750 g)	Serum Amylase: 487 Panc. Amylase: 184 Panc Lipase:786 TG: 2316	

Chee-Chuen Loo [7]	2002	37	G3P2	37	Ranitidine, Heparin, Insulin		SVD		Serum Amylase: 956 TG: 2066	Serum Amylase: 39 TG: 492

J.C. Sleth [8]	2004	28	G2P1	37	Heparin		C/S	Unstable Condition of the Mother	TG: 2316 Cholesterol: 1000 Panc. Amylase: 574 Panc. Lipase: 1310	TG: 100

A. Abu Musa [9]	2006	39	G2P1	28	Plasmapheresis		C/S	A Repeat C/S Delivery	TG: 3810 Panc. Amylase: 525 Panc. Lipase: 3524	TG: 591 Panc. Amylase: 79 Panc. Lipase: 396

Shih-Chang Chuang [10]	2006	28	G1P0	34	Antibiotics, TPN	Pancreatic Necrosectomy, Right Hemicolectomy Ileostomy, Cholecystostomy, Gastrostomy, Feeding Jejunostomy		Unstable Condition of the Mother	TG: 2184 Panc. Amylase: 1365 Panc. Lipase: 533	TG: 319

Alptekin Gürsoy [11]	2006	24	G1P0	37			C/S (3230 g)	Fetal Distress	TG: 10092 Cholesterol: 1159 Panc. Amylase: 367 Panc. Lipase: 797	TG: 143 Cholesterol: 274 Panc Amylase: 23 Panc Lipase: 41

V. Exbrayat [12]	2007	31		33	Plasmapheresis, Heparin		C/S	Fetal Distress	TG: 11300 Cholesterol: 2500 Panc Amylase: 334 Panc Lipase: 168	TG: 1000

Luminita S. Crisan [13] - 1	2008	27	G2P0	35	TPN, Analgesics, Bowel Rest	ARDS	C/S (2653 g)	Fetal Distress		

Luminita S. Crisan [13] - 2	2008	29	G3P1	30	TPN, Analgesics, Bowel Rest	Acute Myocardial Infarction	Forceps– Assisted Vaginal Delivery (1854 g)			

Luminita S. Crisan [13] - 3	2008	34	G3P0	33	TPN, Analgesics, Bowel Rest	ARDS	SVD (2147 g)			

Luminita S. Crisan [13] - 4	2008	23	G1P0	35	TPN, Analgesics, Bowel Rest		C/S (2498 g)	Low BPP		

L. Vandenbroucke [14]	2009	34		37	Heparin, A Low-Fat Diet		C/S (3940 g)	Fetal Distress	TG: 8447	TG: 240

Dilek Altun [15] - 1	2012	27	G1P0	5	Plasmapheresis, Heparin			Termination	TG: 2225 Cholesterol: 470 Panc. Amylase: 959	TG: 278 Cholesterol: 181

Dilek Altun [15] - 2	2012	24	G1P0	34	Plasmapheresis, A Low-Fat Diet		C/S (3100 g)		TG: 2699 Cholesterol: 230 Panc. Amylase: 956 Panc. Lipase: 2580	TG: 570 Cholesterol: 2500 Panc. Amylase: 208 Panc. Lipase: 208

Mindaugas Serpytis [16]	2012	31	G2P0	33	Heparin, Insulin, Plasmapheresis				TG: 1576	TG:183

Kumar Thulasidass [17] - 1	2013	37	G3P0	14	Insulin, Metformin, Fish Oil Therapy	Termination			TG: 1421 Cholesterol: 481 Serum Amylase: 1464	TG: 111 Cholesterol:93

Kumar Thulasidass [17] - 2	2013	24	G1P0	8		ARDS	Spontaneous Abortion		TG: 839 Cholesterol: 300 Serum Amylase: 8962	TG: 57 Cholesterol:77

Rafet Basar [18] - 1	2013	32	G3P0	37	Heparin, Fatty Acids, DF		C/S	Elective	TG: 1400	TG: 380

Rafet Basar [18] - 2		30	G2P1	36	Heparin, Fatty Acids, DF, Plasmapheresis		C/S	Elective	TG: 12000	TG: 758

Ying Hang [19]	2013	31	G2P0	27		Noninvasive Positive Pressure Ventilation (NPPV), Drainage of Chylous Ascites, Peritoneal Lavage, ARDS	C/S (1180 g)	Fetal Distress	TG: 523 Cholesterol:325 Panc. Amylase: 178	TG: Normal Cholesterol: Normal

Bahiyah Abdullah [20]	2014	25	G4P3	8		Diagnostic Laparoscopy, Acute Hemorrhagic Pancreatitis	Spontaneous Abortion		Serum Amylase: 1273	Serum Amylase: 147

Tejal Amin [21]	2014	40	G5P4	18	Insulin	IUMF			TG: 836 Cholesterol: 300	TG: 90

Natasha Gupta [22]	2014	32	G5P4	38	Plasmapheresis	Preeclampsia, Pleural Effusion, Chronic Pericarditis, Retinal Detachment	C/S	Unstable Condition of the Mother	TG: 12.570 Cholesterol: 1067 Panc. Amylase: 1617 Panc. Lipase: 1330	TG: 295 Cholesterol: 179

Fadi Safi [23]	2014	24	G9P8	35	Plasmapheresis		C/S (1720 gr)	Unresponsiveness to Treatment	TG: 2661 Cholesterol: 683 Serum Amylase: 802	TG: 425

Rachel Lim [24]	2015	27	G1P0	33	Insulin, Plasmapheresis	Placental Abruption	SVD		TG: 720 Cholesterol: 90 Panc. Lipase: 504	TG: 41

Ying Liu [24]	2015	30	G1P0	32	Plasmapheresis	Compound Heterozygosity (Glu242Lys and Leu252VaL)	C/S	Fetal Distress	TG: 2160 Cholesterol: 670 Panc. Amylase: 132	TG: 420

Funda Gok [25]	2015	37		31	Insulin, DF	IUMF	SVD		TG: 9742 Cholesterol: 705 Panc. Amylase: 570 Panc. Lipase: 319	TG: 556 Panc. Amylase: 107 Panc. Lipase: 77

Hae Rin Jeon [26]	2016	28	G1P0	23		IUMF, Pancreatic Cells Necrotized, Diabetic Ketoacidosis, Metabolic Acidosis, Cardiac Arrest, EX			TG: 10392 Cholesterol: 1006 Panc. Amylase: 1833 Panc. Lipase: 1863	

Ioanna Polypathelli [27]	2017	38	G2P1	30	Heparin, Fatty Acids, Antibiotics		C/S	Resistant Exaggerated Thrombocytosis	TG: 14440 Cholesterol: 1600 Serum Amylase: 540	TG: 521

Tamanna Chibber [28]	2017	38		11		Cardiac Arrest, EX			TG: >1254 Cholesterol: 648 Panc. Lipase: 1079	

BW: birth weight, G: gravida, P: parity, SVD: spontaneous vaginal delivery, BPP: biophysical profile, TPN: total parenteral nutrition, DF: double filtration apheresis, C/S: cesarean section, TG: triglyceride, ARDS: Adult Respiratory Distress Syndrome, and IUMF: Intra-Uterine Mort Fetus.

Triglyceride and total cholesterol units are calculated in mg/dL. Other units are converted to mg/dL.

Serum Amylase: normal range is between 30 and 110 (U/L) [11].

Pancreatic Amylase: normal range is between 17 and 115 (U/L) [11].

Pancreatic Lipase: normal range is between 13 and 60 U/L (U/L) [11].

TG: normal range is between 50 and 160 mg/dL (mg/dL) [11].

Cholesterol: normal range is between 130 and 230 (mg/dL) [11].

*∗* Highest values.

*∗∗* Lowest values.
